# Complementary feeding practices among mothers and nutritional status of infants in Akpabuyo Area, Cross River State Nigeria

**DOI:** 10.1186/s40064-016-3751-7

**Published:** 2016-12-05

**Authors:** Ekerette Emmanuel Udoh, Olukemi K. Amodu

**Affiliations:** Institute of Child Health, College of Medicine, University of Ibadan, Ibadan, Nigeria

**Keywords:** Breastfeeding, Complementary feeding, Nutritional status, Infant feeding practise, Bottle feeding, Diarrhoea

## Abstract

**Background:**

Malnutrition in infants during weaning has been attributed to inappropriate complementary feeding practices and it underlies more than one-third of child mortality in Nigeria. Thus, addressing the influence of complementary feeding practice on nutritional status may be an important approach to reducing the burden of child malnutrition. This cross-sectional study investigated the association between complementary feeding practices among mothers and nutritional status of their infants in Akpabuyo Local Government Area, Nigeria. The study enrolled 330 mother–child pairs from 10 randomly selected out of 32 Health Facilities in Akpabuyo. Socio-demographic information, child and maternal characteristics were obtained using an interviewer-administered questionnaire. Complementary feeding practices were assessed with World Health Organization infant and young child feeding indicators. Nutritional indicators wasting, underweight and stunting were determined.

**Results:**

Prevalence of timely introduction of complementary feeding among infants aged 6–8 months was 85.4%, minimum dietary diversity rate was 31.5%, and minimum meal frequency 36.7%, the rate of minimum acceptable diet was 7.3%. One-third (33.3%) of the infants were underweight, 26.4%, wasted and 24.6%, stunted. Children who did not receive timely complementary foods had higher odds for wasting (OR 5.15; 95% CI 1.50–17.73). Children who did not receive the minimum dietary diversity had higher odds for underweight than children who received the minimum dietary diversity (OR 2.07; 95% CI 1.17–3.70). Children who did not receive the minimum feeding frequency were more likely to be stunted than their peers who received the minimum feeding frequency (OR 1.57; 95% CI 1.53–4.03).

**Conclusion:**

Sub-optimal complementary feeding predisposed to infant’s malnutrition.

## Significance

**What is already known?** Malnutrition is a significant public health problem in Nigeria. Growth faltering can occur as a result of inadequacy of complementary food in terms of quality, quantity and frequency of meals.

**What this study adds?** The prevalence of malnutrition in the study area was higher than the national average. Sub-optimal complementary feeding practices among mothers were risk factors to malnutrition among children in Akpabuyo. Maternal and child’s ill health was also significant predictor to child malnutrition.

## Background

Optimal infant feeding practice is an important factor in determining growth and development of a child. For healthy mothers exclusive breast-feeding of infants for up to 6 months is recommended. And at this time when breast milk is no longer sufficient to meet the nutritional requirements of the child especially for energy and micronutrients complementary feeding process is expected to begin. In introducing complementary foods feeding behaviour of mothers change and mothers and/or caregivers adopt various feeding practices which may not comply with standards for optimal infant feeding. Some common practices by mothers during the complementary feeding period bother on the types of food given to infants and the adequacy and frequency of feeding. Food restrictions due to cultural practices, unhygienic practice in bottle feeding, food handling/preparation and responsive breast feeding are also issues of concern during the complementary feeding period for the child (Kruger and Gericke 2003).

Malnutrition in children resulting from inadequate feeding and child care and disease is a major public health problem throughout the developing world including Nigeria (Muller and Krawinkel 2005). Malnutrition is one of the principal underlying causes of death for many of the world’s children contributing to more than a third of under-five deaths globally. About 178 million children globally are stunted and Africa has the highest rates (WHO 2012). In Nigeria 41% of children under-5 years of age are stunted, with an increase from 27% at age 6 months to 50% at 23 months which is the period were complementary feeding intensifies. About 23% of children under-5 years are underweight in Nigeria and the prevalence among children aged 6–23 months is 24%; wasting among under-five children is 13%, and 17% among children aged 6–23 months. On the other hand obesity stands at 9% among under-five children (National Population Commision (NPC) and ICF Macro 2009). Generally, the risk of malnutrition in the first 2 years of life has been directly linked with poor breastfeeding and complementary feeding practices of mothers together with high rate of infectious diseases (Arimond and Ruel 2004; Lutter and Rivera 2003; Daelmans and Saadeh 2003). In Nigeria, over 50% infants are given complementary foods before 6 months and these foods are often of poor nutritional value—mostly inadequate in terms of energy, protein and micronutrients such as iron, zinc, iodine and vitamin A (Federal Ministry of Health [FMH] 2005). Also during the complementary feeding period, the frequency of feeding for the child is usually low, while the quantities and qualities of foods given are usually less than that required for the ages of the child. Thus, complementary feeding process has been associated with major changes in both macronutrient and micronutrient intake of children resulting in malnutrition.

The Millennium Development Goals (MDGs) number 4 targeted the reduction of child mortality by two-third by the year 2015. However, from recent report Nigeria’s progress towards reducing child mortality by a two-third since 1990 is off track, with only an average of 1.2% reduction in under-five mortality yearly (FMH and Save the Children 2011). Annually, nearly one million children die in Nigeria before they reach the age of 5 years and globally about 11 million under-five children die. In 2006 there was an estimated 9.5 million deaths of children of under-5 years of age globally and poor nutrition which increases the risk of illness contributed directly or indirectly to more than one-third of these deaths. Undernourished children are regularly prone to measles, malaria, diarrhoea, pneumonia and other illnesses (FMH and Save the Children 2011). Evidence has shown that children who are malnourished and have deficiencies of micronutrient early in life have a lifelong impairment of cognitive and physical development.

Growth faltering as a result of inadequacy of complementary food in terms of quality, quantity and frequency of meals has been reported (Awobgenja and Ugwuona 2010; Kruger and Gericke 2003; Mushaphi et al. 2008). In a study in northern Nigeria on feeding practices and nutritional status of under-five children, Awogbenja and his colleague adduced that mothers who introduced complementary food too early had high incidence of children who were under-nourished (Awogbenja and Ugwuona 2010). Since malnutrition is a significant public health problem in the nation and poor complementary feeding habits of mothers has been linked with poor nutritional status of infants (Awobgenja and Ugwuona 2010; Lutter and Rivera 2003; Arimond and Ruel 2004). Addressing the influence of complementary feeding practice of mothers on nutritional status of children may be an important approach towards reducing the burden of child malnutrition. Therefore, this study describes the complementary feeding options of mothers and its association with the nutritional status of their infants in a rural setting. The findings of the study will assist programme implementers and stakeholders make evidence-based decision in the improvement of children’s health by promoting better feeding practices to reduce the prevalence of malnutrition in Nigeria particularly in the vulnerable first year of life.

## Methods

### Study setting

This cross-sectional survey was conducted in Akpabuyo Local Government Area in Cross River State (CRS) Nigeria. Apabuyo has a population of 360,000 people and is located in the Southern Senatorial District with headquarters at Ikot Nakanda (CRS Government 2012). Akpabuyo Local Government Area lies between latitude 4°5′ and 5°40′ and longitude 8°25′ and 8°32′East. It lies within the vegetation belt of southern Nigeria and shares the Atlantic coastline with Bakassi to the East and the Republic of Cameroon to the West. The Local Government Area measures approximately 28.5 km^2^ and is predominantly an agricultural area. The major languages spoken are Efik and English, while all the major ethnic groups share a common cultural and ancestral heritage. The people of Akpabuyo are predominantly farmers, traders and fishermen. Data collection for this study was done between April and May which is the beginning of the rainy season and the commencement of a new planting season in the area.

Quantitative data on complementary feeding practices of mothers and nutritional status of their infants were collected using an interviewer administered questionnaire. A total of 10 out of 32 health facilities in the area were randomly selected for use in the study. Sample size calculation was done using Cochran formula (Cochran 1963):

$${\text{n}} = {\text{Z}}^{2} {\text{pq}}/{\text{d}}^{2} ,$$

where n is sample size, Z is 1.96 (the standard normal deviate), p equals 0.73, derived from an estimate of proportions of children age 6–9 months who received complementary food in Nigeria in 2008 (NPC and ICF Macro 2009), q is 1 − p, and ‘d’ is the desired level of precision. A 10% non-response rate and an equal number of participants per health centre were considered; this resulted in a total of 330 mothers with their infants in the age group 6–11 months who were enrolled in this study. A total sampling of 33 eligible mother/child pair from each health facility was done.

The WHO infant and young child feeding questionnaire was used to elicit information on complementary feeding practices (WHO 2010b). A translated version of the pre-tested questionnaire into the local language Efik was carried along with the English version by trained assistants to enable easy communication and recording of data unto the English version. In addition, information on socio-demographic characteristics, maternal and child characteristic were collected. Anthropometric measurement of infants was done to assess nutritional status by measuring weight and height. Weight measurements were done using the Docbel Bruan Baby weighing scale (Docbel Group of Industries, New Delhi, India) and the reading taken to the nearest 0.1 kg. The weighing scale came as a standardized instrument from the manufacturer, however, it was validated by weighing a known weight object to confirm it corresponds with the reading. Height of infants was measured to the nearest 0.1 cm using a measuring board. The dependent variables weight and recumbent length of the children were used to compute the nutritional status indices; weight-for-age (WFA), weight-for-high (WFH) and Height for age (HFA) expressed in z scores using the 2006 WHO child growth standard reference value (Onis 2007).

### Measures

#### Complementary feeding indicators

To assess complementary feeding practices of infants this study adapted the indicators that have been developed due to current recommendations for feeding children according to UNICEF/WHO (WHO 2010a).

#### *Introduction of complementary feeding* is the proportion of infants 6–8 months of age who receive solid, semi-solid or soft foods

*Minimum dietary diversity* is the proportion of children 6–11 months of age who receive foods from 4 or more food groups. The food group is a score created by adding seven groups of food consumed in the past 24 h. Each food group is given a score of one if food consumed. The food group include; grains, roots, tubers; legumes and nuts; dairy products; flesh foods (meat, fish, poultry, organs; eggs; vitamin A-rich fruits and vegetables, and other fruits and vegetables (WHO 2010a).

*Minimum meal frequency* implies the proportion of breastfed and non-breastfed children 6–11 months of age who receive solid, semi-solid, or soft foods (also including milk feeds for non-breastfed children) the minimum number of times or more,

*Minimum acceptable diet* is estimated as the proportion of children 6–11 months of age who receive a minimum acceptable diet (apart from breast milk).

*Bottle feeding* indicates the proportion of children 6–11 months of age who are fed with a bottle; while;

*Milk feeding frequency for non*-*breasted child* is proportion of non-breastfed children 6–11 months of age who receive at least 2 milk feeding.

#### Anthropometry

Weight and height of infants was measured for computing nutritional indicators; [weight-for-height (WFH), weight-for-age (WFA) and height-for-age (HFA) z-scores were determined using WHO anthro v3.2.2. Children were classified as normal (z-score: −2.0 to 2.0), wasted (WFH z-score: < −2.0), underweight (WFA z-score: < −2.0) and stunted (HFA z-score: < −2.0) (Onis 2007).

### Data management and statistical analysis

Data was entered and analysed using the statistical package IBM SPSS Statistics 20.0. Socio-demographic and economic characteristics, health related characteristics, complementary feeding indicators and nutritional status of infants were first analysed by descriptive statistics. Bivariate analysis was done using Chi square to examine association between independent and dependent categorical variables. Multivariate logistic regression employing forward step-wise method was done to model a relationship between predictor variables and nutritional status indicators. Complementary feeding indicators, socio-demographic/economic, mother’s and child’s characteristics and household characteristics were combined in a single model. Statistical significance is determined when p < 0.05.

## Results

### Socio-demographic, maternal and child characteristics

 Table 1 presents the socio-economic characteristics of the respondent mothers. Majority of mothers had a secondary education (48.0%) and primary education (32.1%). A third of the mothers were traders (30.6%) while 27.3% were either housewife or unemployed; only 0.6% was professionals or specialist. Most mothers worked outside home (68.8%) while 31.2% worked at home. Mothers engaged in daily work exceeding 8 h was 52.2% while 46.8% usually worked for about 4–8 h daily; 1.1% were involved in working less than 4 h daily. Majority of mothers had more than three children (44.3%). More than a third of the children were reported to have an episode of diarrhoea in the last 2 weeks (39.1%). Some of the children were reported ill in the previous 1 month preceding the study (48.9%), as 35.5% of the mothers also reported to have been ill the 1 month preceding the survey. More of the socio-demographic information of the participants is shown in Table 1.

**Table 1:** Socio-demographic, maternal and child characteristics of respondents

Characteristics	N	%
Mothers level of education		
None	42	12.6
Primary	107	32.1
Secondary	160	48.0
More than secondary	24	7.3
Mothers occupation		
Professional/Specialist	2	0.6
Civil servant	18	5.4
Farmer	38	11.4
Housewife/Unemployed	91	27.3
Student	46	13.8
Trader	102	30.6
Others	36	10.8
Place of work		
Work at home	59	31.2
Work outside home	130	68.8
Hours of work		
<4	2	1.1
4–8	87	46.8
>8	97	52.2
Mothers income (Naira)		
≤10,000	47	37.6
10,001–20,000	43	34.4
20,001–30,000	18	14.4
30,001–40,000	5	4.0
40,001–50,000	7	5.6
>50,000	5	4.0
Number of people in household		
<5	135	40.7
5–9	187	56.3
>9	10	3.0
Parity		
1	103	31.1
2	82	24.7
≥3	147	44.3
Diarrhoea in the last 2 weeks		
Yes	129	39.1
No	201	60.9
Vitamins, minerals, supplements hospital medicine offered in the previous 7 days		
Yes	163	49.1
No	166	50.0
Don’t know	3	0.9
Child sick in the previous 1 month		
Yes	161	48.9
No	168	51.1
Child’s sickness in the previous 1 month		
Fever	52	35.4
Catarrh/cold/cough	40	27.2
Malaria	26	17.7
Others	29	19.7
Mother sick in the previous 1 month		
Yes	116	35.5
No	211	64.5

### Complementary feeding practices

Result showed that only 25.0% of the mothers started complementary foods at the age of 6 months, while majority had begun complementary feeding before 6 months (73.5%) and the rest delayed more than 6 months (1.5%) (See Table 2 on results of complementary feeding outcome). The proportion of infants 6–8 months of age who were receiving solid, semi-solid or soft foods was 85.4%. The rate of feeding infants the minimum dietary diversity was 31.5%; this rate was lower for infants 6–8 months (25.5%) than infants aged 9–11 months (43.2%). Minimum meal frequency rate was 36.7%; higher among children aged 6–8 months (73.4%) compared with children aged 9–11 months (44.0%). The rate of minimum acceptable diet was 7.3%, which was lower at age 6–9 months (22.9%) compared to infants aged 9–11 months (23.4%). Bottle feeding rate during the previous 24 h was observed to be 35.1%, higher at age 6–9 months than at age 9–11 months (45.4 vs 16.1%). Milk feeding frequency for non-breastfed children was 7.0% and also higher at the age 6–9 months than 9–11 months (7.7 vs 6.7%).

**Table 2:** Complementary feeding indicators and related feeding practices of mothers in Apkabuyo

Characteristics	N	%
Introduction of solid, semi-solid or soft foods		
Met	135	85.4
Not met	23	14.6
Minimum dietary diversity		
Met	101	31.5
Not met	220	68.5
Minimum meal frequency		
Met	200	36.7
Not met	116	63.3
Minimum acceptable diet		
Met	74	23.1
Not met	247	76.9
Bottle feeding		
Yes	112	35.1
No	207	64.9
Milk feeding frequency for non-breastfed child		
Met	3	7.0
Not met	40	93.0
Still breastfeeding		
Yes	283	85.2
No	49	14.8
Age of introducing complementary food		
<6 months	231	73.5
6 months	86	25
>6 months	9	1.5
Separate preparation of child’s food		
Yes	225	67.6
No	108	32.4
Reasons for starting complementary foods		
Hospital instruction	62	18.4
For good growth	27	8.2
Baby was not satisfied	101	30.7
Breast milk was not enough	19	5.8
Always crying	54	16.4
Hungry	35	10.6
Others	31	9.4
First complementary food offered to child		
Infant formula	66	20.0
Custard	113	34.2
Guinea corn (Pap)	123	37.3
Other foods	28	8.5
How complementary food is fed to child		
Bottle with nipple	71	21.5
Spoon	222	67.3
Cup	2	0.6
Hand	34	10.3
Hand washing with soap before feeding child		
Yes	173	52.3
No	158	47.7

A-third of the mothers (30.7%) was of the opinion that their baby was not satisfied with breast milk hence the reason to start complementary food. Some mothers started complementary feeding as a result of instructions given in hospital (18.4%), while 5.8% started complementary feeding because breast milk was not enough; 16.4% reported that baby was always crying as such they began feeding the child with complementary foods. The most common first complementary foods mothers offered to their infants were guinea corn (pap, 37.3%) and custard (34.2%). About 20.0% of mothers gave infant formula alone as the first complementary feed.

Starch based foods were the most consumed food among the children (91.0%); highest consumption of starchy foods was seen among children aged 9–11 months (95.3%) and 89.1% among infants 6–8 months. Overall dairy product consumption rate was 67.6% and higher rate, 78.2%, was observed among infants 6–8 months but 49.2% among infants aged 9–11 months; consumption of vitamin-A rich fruits and vegetables was 50.8% and highly consumed among children aged 9–11 months than children 6–8 months (68.9 vs 40.3%). Other fruits and vegetable consumption was only 10.8%. Fresh foods consumption was 44.1%, higher among children aged 9–11 months than children 6–8 months (61.5 vs 34.1%); consumption of eggs was 9.6% (13.1% among children 9–11 months and 7.6% for children 6–8 months). Legumes and nuts consumption was 18.6%; lower among infants aged 6–8 months than infants 9–11 months of age (16.6 vs 22.1%).

### Nutritional status of infants

Mean and standard deviation values for the anthropometric indicators WHZ, WAZ and HAZ z-scores were −0.67 ± 1.73, −1.10 ± 1.43, −1.02 ± 1.25 respectively. The prevalence of wasting was 26.4% (95% CI 21.5–31.3), overweight and obesity was 5.7% (95% CI 3.1–8.3), underweight was 33.3% (95% CI 28.1–38.5) stunting was 24.6% (95% CI 19.8–29.4). Wasting was more common among males compared with females (34.1 vs 18.7%) (Table 3), underweight among males was 44.9 and 21.7% among females. Stunting was 28.7% for males and 20.5% among females. Overweight was less common among males compared with females (4.2 vs 7.2%). According to age of infants wasting was more prevalent among infants in the age group 9–11 months old (27.0%) than infants aged 6–8 months (26.3%), underweight was common among the age group 9–11 months than 6–8 months (34.2 vs 32.9%), likewise, stunting was more prevalent among 9–11 months old infants than 6–8 months (34.2 vs 19.7%).

**Table 3:** Prevalence of malnutrition among infants by sex

Indicator	Males	Females	All
	%	%	% (95% CI)
Wasting (WFH < −2 z-score)	34.1	18.7	26.4 (21.5–31.3 95% CI)
Severe wasting (WFH < −3 z-score)	10.2	2.4	6.3 (3.5–9.1 95% CI)
Overweight and obesity (WFH > +2 z-score)	4.2	7.2	5.7 (3.1–8.3 95% CI)
Underweight (WFA < −2 z-score)	44.9	21.7	33.3 (28.1–38.5 95% CI)
Severe underweight (WFA < −3 z-score)	12.0	2.4	7.2 (4.9–10.0 95% CI)
Stunting (HFA < −2 z-score)	28.7	20.5	24.6 (19.8–29.4 95% CI)
Severe stunting (HFA < −3 z-score)	4.8	2.4	3.6 (1.5–5.8 95% CI)

### Bivariate associations between infant nutritional status and complementary feeding indicators

Association between infant nutritional status indicators and complementary feeding indicators are presented in Table 4. There was a significant association (p < 0.05) between timely introduction of solids with the nutritional status indicator wasting. Wasting was observed to be more prevalent (39.1%) among children who did not receive timely solid, semi-solid or soft foods compared with children who received solid, semi-solid or soft foods (24.4%).

**Table 4:** Wasting, underweight and stunting of infants by complementary feeding indicators, socio-demographic and maternal and child characteristics

Complementary feeding indicators	Nutritional status of infants 6–11 months								
	Wasting			Underweight			Stunting		
	%	Total	*p*	%	Total	*p*	%	Total	*p*
Introduction of solid, semi-solid or soft foods									
Met	50.0	129	0.032	32.6	135	0.835	20.0	135	0.043
Not met	25.6	18		30.4	23		39.1	23	
Minimum dietary diversity									
Met	25.0	96	0.387	23.8	101	0.011	16.8	101	0.042
Not met	29.8	208		38.2	220		27.3	220	
Minimum meal frequency									
Met	29.2	192	0.839	34.0	200	0.931	20.5	200	0.036
Not met	28.0	107		34.5	116		31.0	116	
Minimum acceptable diet									
Met	27.1	70	0.793	25.7	74	0.038	14.9	74	0.036
Not met	28.8	233		36.0	247		26.7	247	
Bottle feeding									
Yes	26.5	199	0.564	31.2	112	0.469	25.0	112	0.791
No	29.6	102		35.2	207		23.7	207	
Milk feeding frequency for non-breastfed child									
Met	0.0	2	0.434	0.0	3	0.356	33.3	3	0.584
Not met	23.7	38		22.5	40		20.0	40	
Hand washing with soap before feeding child									
Yes	20.8	173	0.029	28.9	173	0.062	25.4	173	0.574
No	32.9	158		38.6	158		22.8	158	
Diarrhoea in the last 2 weeks									
Yes	36.4	129	0.016	38.0	129	0.151	22.5	129	0.550
No	20.4	201		30.3	201		25.4	201	
Vitamins, minerals, supplements hospital medicine offered in the previous 7 days									
Yes	26.4	163	0.283	35.0	163	0.640	23.3	163	0.674
No	26.5	166		32.5	166		25.3	166	
Child sick in the previous 1 month									
Yes	29.8	161	0.187	41.0	161	0.004	23.6	161	0.865
No	23.8	168		26.2	168		24.4	168	
Mother sick in the previous 1 month									
Yes	19.8	116	0.060	30.2	116	0.369	31.0	116	0.004
No	29.9	211		35.1	211		20.9	211	
Child ever immunised									
Yes	26.3	327	0.931	33.3	327	1.000	24.2	327	0.670
No	33.3	6		33.3	6		16.7	6	
Mother’s age									
<20	17.5	40	0.205	25.5	40	0.067	22.5	40	0.751
20–30	25.2	218		30.7	218		22.5	218	
>30	36.5	63		44.4	63		27.0	63	
Marital status									
Single	16.5	19	0.000	30.4	79	0.712	24.1	79	0.729
Married	29.6	250		34.4	250		24.0	250	
Separated	50.0	2		50.0	2		0.0	2	
Mother’s level of education									
None	31.0	42	0.750	40.5	42	0.199	23.8	42	0.922
Primary	29.0	107		38.3	107		26.2	107	
Secondary	25.0	160		30.0	160		22.5	160	
More than secondary	16.7	24		20.8	24		25.0	24	
Mother’s occupation									
Professional/Specialist	0.0	2	0.000	0.0	2	0.001	0.0	2	0.401
Civil servant	11.1	18		5.6	18		5.6	18	
Farmer	44.7	38		57.9	38		57.9	38	
Housewife/Unemployed	19.8	91		29.7	91		29.7	91	
Student	21.7	48		32.6	46		32.6	46	
Trader	33.3	102		38.2	102		38.2	102	
Others	19.4	36		19.4	36		19.4	36	
Place of work									
Work at home	39.0	59	0.272	32.2	59	0466	15.3	59	0.148
Work outside home	29.2	130		37.7	130		24.6	130	
Mothers income (Naira)									
≤10,000	44.7	47	0.000	48.9	47	0.008	23.4	47	0.195
10,001–20,000	32.6	43		37.2	43		27.9	43	
20,001–30000	5.6	18		11.1	18		5.6	18	
30,001–40000	20.0	5		0.0	5		0.0	5	
40,001–50,000	14.3	7		14.3	7		0.0	7	
>50,000	0.0	5		0.0	5		20.0	5	
Number of people in household									
<5	26.7	135	0.447	25.9	135	0.069	24.4	135	0.890
5–9	26.7	187		38.0	187		23.2	187	
>9	10.0	10		40.0	10		24.2	10	
Parity									
1	20.0	105	0.132	23.8	105	0.003	19.0	105	0.219
2	21.7	85		27.7	83		22.9	83	
≥3	33.3	144		43.1	114		28.5	144	
Child’s gender									
Male	34.7	167	0.006	44.3	167	0.000	28.1	167	0.078
Female	18.1	166		22.3	116		19.9	144	
Child’s age (months)									
6–8	27.0	211	0.652	32.7	211	0.826	19.4	211	0.033
9–11	26.1	122		35.1	122		32.4	122	
Birth order of child									
First	18.8	101	0.002	23.8	101	0.005	20.8	101	0.069
Second	23.1	91		30.8	91		23.1	91	
Third	18.0	50		30.0	50		20.0	50	
Fourth	55.6	27		55.6	27		14.8	27	
>Fourth	37.5	64		45.3	64		37.5	64	

The minimum dietary diversity was significantly associated with underweight. Children who did not receive the minimum dietary diversity were more underweight (38.2%) than children who received the minimum dietary diversity (23.8%). Similarly, children who did not receive the minimum acceptable diet were significantly more underweight (36.0.7%) than their counterparts who received the minimum acceptable diet as recommended (25.7%).

The feeding indicators introduction of solid, semi-solid or soft foods; minimum dietary diversity and minimum meal frequency showed significant association with stunting. Children who were not receiving solid, semi-solid or soft foods at 6–8 months of age were more stunted than children who received complementary foods at 6–8 months (39.1 vs 20.0%); stunting was also more prevalent among infants who were not receiving the minimum dietary diversity (27.3%) relative with infants who received the minimum dietary diversity (16.8%). Infants who did not receive the minimum meal frequency were more stunted (20.5%) compared with children who met the minimum feeding frequency criteria.

Wasting in children was higher among infants whose mothers do not wash hands with soap before feeding the child (32.9%) relative to infants whose mothers washed hands with soap before feeding child (20.8%) (see Table 4). Infants who had diarrhoea 2 weeks to the study had higher prevalence of wasting (36.4%) relative to those who did not have diarrhoea (20.4%). Further analysis showed that of the proportion of infants who were wasted a significant proportion had diarrhoea and their mother did not wash hands with soap before feeding child (50%) (Table 5). Maternal ill health in the month preceeding the survey showed a significant association with child stunting, as 31% children of these mothers were stunted compared with children whose mothers were not ill (20.9%).

**Table 5:** Undernutrition by diarrhoea as function of hand washing with soap before feeding child

Diarrhoea	Hand washing with soap before feeding child	Wasting			Underweight			Stunting		
		%	Total	*p*	%	Total	*p*	%	Total	*p*
Yes	Yes	25.9	58	0.006	26.2	61	0.007	19.7	61	0.442
	No	50.0	64		49.3	67		25.4	67	
No	Yes	20.4	103	0.604	30.0	110	0.865	29.1	110	0.198
	No	23.5	85		31.1	90		21.1	90	

### Multivariate associations for factors predicting infant nutritional status

In the multivariate association using forward stepwise logistic regression process, selected independent variables were combined in a model with wasting, underweight and stunting to determine factors that remained significant after controlling for confounding effects. The following factors; complementary feeding indicators, socio-demographic/economic, mother’s and child’s characteristics and household characteristics were combined in a model. All the models were statistically significant, implying that the model with all the factors included significantly improved prediction of the outcome compared with the constant only model. However, the models that presented an excellent fit as the pseudo R^2^ showed were the adjusted model for underweight and stunting (Tables 6, 7, respectively). The factors that remained significantly associated with wasting in adjusted model were introduction of solid, semi-solid and soft foods and gender of child (see Table 6). Children who did not receive solid, semi-solid and soft foods at 6–8 months were five times more likely to be wasted compared with children who received solid, semi-solid and soft foods (OR 5.15; 95% CI 1.50–17.73, p < 0.05). Female children were less likely to be wasted compared with their male counterparts (OR 0.18; 95% CI 0.06–3.59, p < 0.05).

**Table 6:** Adjusted model for factors related with infant wasting

Predictors	Odds ratio (95% CI)	*p*
Introduction of solid, semi-solid and soft foods		
Met (Reference)	1.00	
Not met	5.15 (1.49–17.73)	0.009
Marital status		
Single (Reference)	1.00	
Married	2.55 (0.80–7.69)	0.110
Gender of child		
Male (Reference)	1.00	
Female	0.18 (0.06–0.46)	0.000
Washing hands with soap		
Yes (Reference)	1.00	
No	1.56 (0.64–3.59)	0.286
Diarrhoea in the last 2 weeks		
No (Reference)	1.00	
Yes	1.81 (0.76–4.31)	0.180
Mother’s income		
≤30,000 (Reference)	1.00	
≥30,000	1.06 (0.37–3.22)	0.913
Maternal occupation		
Housewife (Reference)	1.00	
Full/par-time job	0.59 (0.19–1.85)	0.374

Model Chi-Square = 20.562, *p* = 0.021, Negelkerk R^2^ = 0.423

**Table 7:** Adjusted model for factors related with infant underweight

Predictors	Odds ratio (95% CI)	*p*
Minimum dietary diversity		
Met (Reference)	1.00	
Not met	2.07 (1.17–3.70)	0.013
Gender of child		
Male (Reference)	1.00	
Female	0.34 (0.27–0.61)	0.000
Mother’s income		
≤30,000 (Reference)	1.00	
≥30,000	0.98 (0.48–1.96)	0.951
Maternal occupation		
Housewife (Reference)	1.00	
Full/par-time job	1.47 (0.74–2.58)	0.262
Parity		
1 (Reference)	1.00	0.181
2	0.71 (0.07–7.69)	0.784
≥3	2.27 (0.25–10.0)	0.459
Birth order of child		
First (Reference)	1.00	0.557
Second	1.00	0.685
Third	1.61 (0.15–12.66)	0.844
Fourth	0.78 (0.07–8.33)	0.603
>Fourth	1.85 (0.18–13.00)	0.921
Child sick in the previous 1 month		
No (Reference)	1.00	
Yes	1.80 (1.07–3.01)	0.027

Model Chi-Square = 29.822, *p* = 0.001, Negelkerk R^2^ = 0.678

Two factors, minimum dietary diversity and gender of child remained significantly associated with underweight after controlling for maternal, child and household characteristics (Table 7). Children who did not receive the minimum meal diversity were two times more likely to be underweight compared with children who received the minimum meal diversity (OR 2.07; 95% CI 1.17–3.70, p < 0.05). Female children were less likely be underweight compared with male children (OR 0.34; 95% CI 0.27–0.61, p < 0.05).

Infants who did not receive the minimum dietary diversity and minimum meal frequency were significantly more likely to be stunted; (OR 2.17; 95% CI 1.43–4.20, p < 0.05) and (OR 1.57; 95% CI 0.53–4.03, p < 0.05) respectively. Maternal illness in the previous 1 month remained significantly associated with stunting (Table 8). Children of mothers who were ill were two times more likely to be stunted compared with children whose mothers were not ill in the previous 1 month (OR 2.35; 95% CI 1.06–5.22, p < 0.05) (Table 7).

**Table 8:** Adjusted model for factors related with infant stunting

Predictors	Odds ratio (95% CI)	*p*
Introduction of solid, semi-solid and soft foods		
Met (Reference)	1.00	
Not met	1.56 (0.42–5.71)	0.499
Minimum dietary diversity		
Met (Reference)	1.00	
Not met	2.17 (1.43–4.20)	0.043
Minimum meal frequency		
Met (Reference)	1.00	
Not met	1.57 (1.53–4.03)	0.035
Child’s age (months)	0.63 (0.33–1.20)	0.168
Mother sick in the previous 1 month		
No (Reference)	1.00	
Yes	2.35 (1.06–5.22)	0.036

Model Chi-Square = 27.655, *p* = 0.011, Negelkerk R^2^ = 0.585

## Discussion

In healthy infants introducing complementary feeding must be delayed till 6 months as WHO recommends exclusive breastfeeding for this period to confer several benefits to the infant and mother. Studies have demonstrated that early introduction to solid foods is a risk factor for infection, early cessation of breastfeeding and increased consumption of fatty or sugary foods at 1 year of age (Grummer-Strawn et al. 2008). Our result showed that 2.3% mothers introduced complementary foods to infants as early as the first month of life and 12.9% at 2 months. Overall, 73.5% of the mothers in Akpabuyo introduced complementary food to their infant before the recommended age of 6 months. This high frequency is similar with studies in Nasarawa, Nigeria, where 69.0–82% mothers are reported to have introduced complementary foods before 6 months (Awogbenja and Ugwuona 2010). In another study in Pakistan, a developing country like Nigeria, lesser prevalence (21%) of starting complementary foods at an inappropriate time has been recorded (Liaqat et al. 2006). Poor practice of early introduction of complementary foods may be due to the wrong perception mothers have about feeding breast milk alone for the recommended duration of 6 months. In fact we observed that majority of mothers in the study area (30.7%) believed that the baby was not satisfied with breast milk as such they felt complementary feeding should commence.

Introduction of semi-solid, solid of soft foods at age 6–8 months was higher than the national prevalence of 72.8% from the 2008 National Demographic Health Survey (NDHS) (NPC and ICF Macro 2009). The high rate of receiving complementary foods at 6–8 months in the present study is encouraging, however, not surprising since a large percentage of mothers in Akpabuyo begun feeding complementary foods to their infants before the age of 6 months which is inappropriate. The finding of our study is consistent with a study by Marriott et al. (2011) in 14 low-income countries including Nigeria. Their study indicated that the rate of infants 6–8 months receiving complementary foods ranged between 50% in Ethiopia to 90% in Tanzania. In another study using DHS data and National Family Health Survey data from five South Asian countries, the rate of introduction of solid ranged from 34% in Pakistan to 84% in Sri Lanka (Senarath et al. 2012).

The minimum dietary diversity for all infants 6–11 months was low at 31.5%. Poor diet diversity is a common practice in poor populations because the main complementary diets are mainly starch based staples, with few animal products and vegetables (Arimond and Ruel 2004). Conversely, results indicated the high level at which starchy foods such as grains, roots and tuber foods are being consumed in Akpabuyo compared with more expensive foods like legumes and nuts, eggs and flesh foods which are rich in protein. Many mothers are not buoyant enough to afford expensive animal and vegetable products. Other reasons such as cultural, religious, social, and family influences can limit the types of diets many mothers offer their infants (Odebiyi 1989; Manzoor et al. 2009). Consumption of flesh foods which are rich in iron was encouraging, since iron deficiency is linked with 134,000 deaths annually and regular intake of iron rich foods has been reported to result in the reduction of the prevalence of anaemia among young children from 10 to 40% in less than a year (World Food Programme and UNICEF 2006).

Dietary diversity in the present study was low. Ajani (2010) reported that in Nigeria dietary diversity is generally poor. In the study which assed dietary diversity in six Nigerian states and employing a total of 14 food group score, 83% of the children under the age of 5 months received 5–9 food groups. In the present study dietary diversity employed a range of four food groups and above from a score of seven food groups (WHO 2010a). In another study in Zambia and Ethiopia (Disha et al. 2012) which used the current indicator as employed in our study, a low rate for minimum dietary diversity was observed in Ethiopia (7%) and Zambia (37%). This data suggest a preponderance of low rate of minimum dietary diversity across low income countries. Minimum feeding frequency was also observed to be low. Low feeding frequency has been attributed to fewer amounts of time mothers put in for care for their infants due to their workload. Many mothers work outside the home and often go out for many hours and sometimes food may not be prepared which can be offered to the child by other family member. Number of meals offered to the child may also depend on the income of the family and who is mainly in control of resources in the family.

The prevalence of minimum acceptable diet for infants aged 6–11 months was low (23.1%). To meet the minimum acceptable diet mothers need to be able to feed their children diverse diet and the recommended number of meals. This, however, can be difficult for many mothers to achieve in poor societies and who have low economic capacity to secure food for their household.

Although majority of mothers (64.9%) did not feed their children with bottle with a nipple, a reasonable proportion (35.1%) used bottle with a nipple to feed their infants. As a hygiene concern the practice of feeding infants with feeding bottles has been discouraged by WHO because of the difficulties in maintaining feeding bottles that are free of pathogen which can cause infections. Studies have reported high incidence of diarrhoea in the second phase of infancy due to poor hygiene (Bern et al. 1992). A more appropriate means of feeding infants is by the use of spoons or cups. In this study most of the mothers 67.2% fed their infants appropriately using a spoon which is encouraging. The proportion of non-breastfed infants 6–11 months who received at least two milk feedings in the previous day preceding the study was alarmingly low (7%) considering the fact that these infants are not receiving breast milk. Consumption of diary product generally for all children in this study, however, was seen to be quite high 67.6%.

The overall prevalence of wasting reflecting acute malnutrition among the infants aged 6–11 was very high according to WHO criteria (Onis 2003). However, severe wasting prevalence was low. Overweight and obesity were also low. Underweight which reflects chronic or acute malnutrition among the infants was at a critical level, while severe underweight was low. Stunting which reflects chronic malnutrition was at a medium level while severe stunting was rare. The result indicated that wasting was very high among the males compared with females; underweight prevalence was also very high for both males and females. Prevalence of stunting was at medium level for both males and females. Overweight was less among males compared with females. Higher prevalence of wasting and underweight among males over females have been previously reported (Adeladza 2010; Disha et al. 2012). Results of our study show an apparent increase in malnutrition in infants as children increase in age from 6 months (Marriott et al. 2011; Kumar et al. 2006; NPC and ICF Macro 2009). Wasting increased slightly from 26.3% among infants aged 6–8 to 27% among infants 9–11 months. Underweight and stunting also increased with age of the child. As mothers decrease breastfeeding and complementary feeding intensifies, and as the infant ages and the demands for energy and nutrient increase, the quality of complementary foods offered may not be adequate to maintain the better nutritional status the child acquired earlier when breastfeeding was mainly offered with little complementary foods.

Mothers who introduced timely complementary feeding had children with better WHZ than mothers who had not introduced complementary feeding. Similar finding has been observed before in Zambia (Disha et al. 2012). For infants 6–8 months in the Zambia study, consumption of complementary food was positively associated with WHZ score. Result also confirms that introduction of complementary food is significantly associated with stunting after controlling for maternal characteristics, household and child’s characteristics. Children who received semi-solid, solid and soft foods at 6–8 months were less stunted (80%) compared with those who did not receive complementary foods (60.9%), this finding is also comparable with another studies (Marriott et al. (2011). The present study indicates that failure in the consumption of complementary foods have the likelihood of resulting in wasting and later stunting of infants: because the indicator introduction of semi-solid, solid or soft food at 6–8 months does not measure the quality or frequency of diets for infants, for wasting and stunting to result due to delay in introduction of complementary food it is obvious that energy need of the young child has not been met.

Our study identified association between underweight and dietary diversity. The association remained after controlling for several factors such as gender of child, mother’s income and parity. Children who received the minimum dietary diversity were less likely to be underweight compared with children who did not receive the minimum dietary diversity. Also, children who received the minimum dietary diversity were less likely to be stunted compared with children who did not receive the minimum dietary diversity. This observation is in line with previous findings by Marriott et al. (2011). In another study by Arimond and Ruel (2004), the authors demonstrated that children aged 6–23 months who received diverse diet had better HAZ compared with children who did not receive diverse diet.

It was observed that the minimum meal frequency was only associated with stunting but not wasting and underweight and remained significant at multivariate level. Children who received the minimum meal frequency were less stunted compared with children who did not receive the minimum meal frequency. Disha et al. (2012) showed very weak association between feeding frequency and stunting and underweight in Ethiopia. Marriott et al. (2011) in their study, however, found an association between feeding frequency with underweight, but not with stunting. The association between feeding frequency and stunting in the present study is expected as the indicator is mutually inclusive of feeding of complementary food at 6–8 months which equally showed similar association with stunting.

Child Illness still emerged as a predictor of underweight in infants even after controlling for food diversity and gender. This finding support the fact that diseases and infections have direct effect on the nutritional status of children since it affects dietary intake and utilisation. Maternal illness was a significant predictor to child stunting. This probably may be due to reduced capacity to child care, reduced breast feeding and feeding attention that may arise during the period of mother’s ill health. In Nigeria, although mothers are the main care providers for children, sometimes mothers ignore their health and their own nutrition, making not only themselves vulnerable to undernutrition, but their children too. There is evidence that adequate maternal knowledge on child feeding is pertinent to better health and development of the child. Although the present study did not investigate the impact of mothers’ knowledge on child feeding in the study area and its consequence with children’s malnutrition, many mothers in Cross River State lack the knowledge on adequate child feeding (Jemide et al. 2016). It is also important to note that poverty is widespread in many poor societies and the problem of food insecurity as its corollary cannot be overridden as one of the major causes, in addition to lack of maternal knowledge on feeding, of malnutrition among children in the study area.

While this study has provided relevant facts on child malnutrition in the area it was not devoid of some limitations. The limitations included reporting and recall bias a problem common to cross-sectional surveys, particularly for retrospective data relying on memory of past events. This problem in reporting and recall was limited as much as possible by probing respondents to further give details of their responses.

## Conclusion

The prevalence of malnutrition in Akpabuyo based on wasting, underweight and stunting were of public health significance. Children who received timely complementary foods had normal status based on weight-for-height than children who did not receive timely complementary foods even after controlling for maternal, child and health factors. Dietary diversity was a predictor of better weight-for-age. Children who received the minimum feeding frequency had the likelihood of becoming less stunted than their peers who did not receive the minimum feeding frequency. Maternal illness was a significant predictor to child stunting.

## Recommendations

1. Intervention efforts to improve nutritional status of infants through nutrition and educational inputs should emphasise optimal infants feeding practices.
2. Interventional programmes should target poorer household and mothers with lower educational level to improve complementary feeding practices of mothers.
3. Developmental programmes should focus on empowering women in the community by improving of household income through creation of employment and assess to credit facilities that will enable women engage in sustainable means of livelihood.
4. There is need for support for mothers to commit to continued and adequate care to older children without discrimination to gender to protect children from deteriorating nutritional status as they grow.
5. Caregivers and mothers should be educated on maintenance of hygienic conditions of the home and during feeding of the child to prevent diarrhoea and illness, and on the management of diarrhoea.
6. There is need for promotion of women’s health and nutrition as a strategy that will benefit child nutritional status.
